# The Interplay between Inflammation, Coagulation and Endothelial Injury in the Early Phase of Acute Pancreatitis: Clinical Implications

**DOI:** 10.3390/ijms18020354

**Published:** 2017-02-08

**Authors:** Paulina Dumnicka, Dawid Maduzia, Piotr Ceranowicz, Rafał Olszanecki, Ryszard Drożdż, Beata Kuśnierz-Cabala

**Affiliations:** 1 Department of Medical Diagnostics, Jagiellonian University Medical College, Medyczna 9, 30-688 Kraków, Poland; paulina.dumnicka@uj.edu.pl (P.D.); ryszard.drozdz@uj.edu.pl (R.D.); 2 Department of Anatomy, Jagiellonian University Medical College, Kopernika 12, 31-034 Kraków, Poland; dawid.maduzia@uj.edu.pl; 3 Department of Physiology, Jagiellonian University Medical College, Grzegórzecka 16, 31-531 Kraków, Poland; 4 Department of Pharmacology, Jagiellonian University Medical College, Grzegórzecka 16, 31-531 Kraków, Poland; rafal.olszanecki@uj.edu.pl; 5 Department of Diagnostics, Chair of Clinical Biochemistry, Jagiellonian University Medical College, Kopernika 15A, 31-501 Kraków, Poland; mbkusnie@cyf-kr.edu.pl

**Keywords:** acute pancreatitis, coagulation, endothelial injury, inflammation, laboratory markers

## Abstract

Acute pancreatitis (AP) is an inflammatory disease with varied severity, ranging from mild local inflammation to severe systemic involvement resulting in substantial mortality. Early pathologic events in AP, both local and systemic, are associated with vascular derangements, including endothelial activation and injury, dysregulation of vasomotor tone, increased vascular permeability, increased leukocyte migration to tissues, and activation of coagulation. The purpose of the review was to summarize current evidence regarding the interplay between inflammation, coagulation and endothelial dysfunction in the early phase of AP. Practical aspects were emphasized: (1) we summarized available data on diagnostic usefulness of the markers of endothelial dysfunction and activated coagulation in early prediction of severe AP; (2) we reviewed in detail the results of experimental studies and clinical trials targeting coagulation-inflammation interactions in severe AP. Among laboratory tests, d-dimer and angiopoietin-2 measurements seem the most useful in early prediction of severe AP. Although most clinical trials evaluating anticoagulants in treatment of severe AP did not show benefits, they also did not show significantly increased bleeding risk. Promising results of human trials were published for low molecular weight heparin treatment. Several anticoagulants that proved beneficial in animal experiments are thus worth testing in patients.

## 1. Introduction

Acute pancreatitis (AP) is the most common cause of acute hospital admissions among gastrointestinal diseases, with the incidence of about 10–100 per 100,000 population [1,2,3]. Increasing incidence has been recently reported in the USA and many European countries [1,4]. The disease is characterized by the spectrum of severity: most cases are mild and self-limiting; however, about 30% of cases are classified as moderately severe, and about 10% as severe according to the 2012 revision of the Atlanta classification [5,6]. Organ failure is the main determinant of severity and the main cause of early mortality, while secondary infections, including infected pancreatic necrosis and sepsis, are responsible for the late deaths [5]. Overall mortality in AP is about 3%–6%, whereas in severe AP (SAP), it reaches 30% [1,6]. The high mortality is associated with the lack of specific treatment; however, a decrease in mortality has been achieved thanks to improved intensive care and less invasive surgical management in severe cases [1,6,7]. As indicated in current clinical guidelines [7], early (within first 24 h from admission) and adequate fluid resuscitation decreases the rates of persistent systemic inflammatory response syndrome (SIRS), organ failure and mortality.

Although the etiology of AP is complex [8], the two most common causes are biliary tract diseases and excessive alcohol consumption [6]. Premature activation of digestive enzymes (most importantly, trypsinogen into trypsin) within acinar cells is the key event in early pathogenesis of AP, leading to destruction (autodigestion) of the pancreas [9,10]. Unconjugated bile acids and fatty acid ethyl esters (the products of non-oxidative alcohol metabolism) cause mitochondrial injury and sustained increase in intracellular Ca^2+^ concentrations in acinar cells, leading to inhibition of zymogen secretion and premature activation of digestive enzymes [11,12]. Recent studies have shown that acinar cells form a functional unit with ductal cells. Low doses of bile acids or alcohol cause increased secretion of bicarbonate-rich fluid by pancreatic ductal cells that may protect acinar cells from the contact with toxic substances. To the contrary, high concentrations of unconjugated bile acids and alcohol inhibit secretion of bicarbonate-rich fluid by pancreatic ducts. Thus, the initial events in AP involve both ductal and acinar cells [13,14].

Irrespective of the causative factor, acinar injury is associated with early inflammatory reaction within the pancreas, characterized by nuclear factor κB (NFκB) activation and cytokine production in acinar cells, at least partially independent of trypsinogen activation [15,16,17]. As a consequence, inflammatory cells, including neutrophils and monocytes, are activated and recruited to the pancreas, exaggerating the damage of the gland as well as the inflammation [18]. In particular, neutrophils are the source of tissue-degrading enzymes, reactive oxygen species, and further inflammatory cytokines [19]. Most recently, the formation of neutrophil extracellular traps has been documented within pancreatic ducts, which enhances premature activation of trypsinogen [20,21]. Another consequence of local inflammation is vascular injury within the pancreas, manifesting as endothelial activation and endothelial injury, increased vascular permeability, activation of coagulation, and increased leukocyte rolling, sticking and transmigration to pancreatic tissue [22,23]. In mild AP, the inflammatory response is local and self-limiting, whereas in SAP, excessive systemic inflammation develops. The levels of proinflammatory cytokines and acute phase proteins in systemic circulation correlate positively with the severity of AP [17,18,24]. In SAP, trypsin, damage-associated molecular patterns, and proinflammatory cytokines released from the inflamed pancreas lead to systemic vascular injury with vascular leak syndrome and cardiovascular, kidney and lung failure [22,25]. Systemic endothelial dysfunction may also manifest itself as diffuse activation of coagulation, with clinically significant thrombotic complications observed in a part of patients with SAP [26,27].

Despite recent progress in understanding the early events in AP, more research is needed to enable faster and more accurate prediction of a severe course of the disease as well as more specific and better targeted treatment [3]. At present, prediction of SAP is based on clinical assessment at admission and during the treatment [7]. The laboratory markers of trypsinogen activation or inflammation [24], the severity scores based on computer tomography imaging, and the multi-parameter severity scores such as bedside index of severity in AP (BISAP) or acute physiology and chronic health evaluation (APACHE) have been proposed for prediction of SAP [28,29]. While they are helpful, they are far from perfect. The biomarkers associated with systemic vascular injury may prove a useful alternative or supplementation.

The purpose of the review is to summarize current evidence regarding the interplay between inflammation, coagulation and endothelial dysfunction in the early phase of AP. Practical aspects are underscored, such as the possibilities to use the markers of endothelial dysfunction and activated coagulation in early prediction of SAP, as well as the possibilities of targeting coagulation-inflammation interactions in the treatment of SAP.

## 2. Interrelations between Coagulation and Inflammation

Coagulation and inflammation clearly show reciprocal connections. Activation of coagulation leads to stimulation of inflammatory mechanisms [30,31]. The contact of factor VII with tissue factor (TF) is the main trigger for activation of coagulation. The complex formed by TF and activated factor VII (VIIa) in the presence of factor X stimulates protease-activated receptors (PARs) [32,33]. Also thrombin as a serine protease activates PARs [34]. PARs are expressed by platelets and by numerous immune cells such as monocytes, lymphocytes, macrophages, dendritic cells and mast cells, as well as by endothelial cells [35]. Once thrombin affects PARs present on the membranes of platelets, platelet activation occurs with shape change and the release of granules’ content, including adenosine diphosphate, serotonin, thromboxane and chemokines [30]. Activation of platelets occurs as well in the platelet phase of local hemostasis. Platelet stimulation leads to the release of soluble ligand for CD40 receptor (sCD40L) [36,37]. CD40 molecule belongs to the tumor necrosis factor (TNF) receptor family. Soluble CD40L causes stimulation of TF production and the release of proinflammatory cytokines [38]. Acting on vascular endothelial cells, sCD40L, in addition to chemokine release, causes expression of adhesion proteins, leading to rolling and sticking of leukocytes to the vascular endothelium, their subsequent migration through the vascular wall and inflammatory infiltration of tissues [39]. Moreover, thrombin may directly stimulate vascular endothelial cells, leading to increased vascular permeability, expression of the adhesion proteins such as P-selectin, release of von Willebrand factor (vWF), as well as increased production and release of cytokines [30].

The role of thrombin in the activation of inflammatory process is also related to its chemotactic activity on monocytes, mitogenic activity on lymphocytes and stimulating effect on the production and release of proinflammatory cytokines, particularly TNF-α, interleukin (IL)-1β and IL-6 [34]. In addition, thrombin is capable of activating the complement system that plays an important role in humoral innate immune response and modifies the specific immune response [40]. Thrombin can cause formation of complement fragment C5a, an anaphylatoxin not related to the classic, alternative or lectin pathway of complement activation [41].

Other serine proteases of the coagulation system, including a complex formed by TF and activated factor VII, act directly on endothelial cells, macrophages and monocytes stimulating their proinflammatory mechanisms, such as production of free radicals and expression of adhesion proteins [30,42]. Also, fibrin formed as a result of activated coagulation as well as fibrin/fibrinogen-degradation products act in a proinflammatory manner via activation of leukocytes [43,44] and influence the vascular endothelial cells, which are stimulated to produce proinflammatory cytokines [45].

The relationships between coagulation and inflammation are two-way and are based on positive feedbacks. Therefore, development of inflammation leads to activation of coagulation [30,31]. Trauma, tissue injury, hypoperfusion, hemodilution, hypothermia and acidosis induce acute posttraumatic coagulopathy. Inflammation is an important causative factor [46]. The inflammatory process activates the coagulation system, reduces the activity of natural anticoagulants and disturbs functioning of the fibrinolysis system, leading to (microvascular) thrombosis [31]. Cytokines play an important role in activation of the coagulation system and formation of fibrin, through their action in the extrinsic coagulation pathway, i.e., induction of TF expression on endothelial cells and monocytes [47,48].

Recently, the contact system (or intrinsic pathway) has garnered increasing interest. Although the deficiencies of factor XII (Hageman’s anomaly), plasma prekallikrein or high-molecular weight kininogen do not lead to bleeding disorders, activation of the contact system has been implicated in thrombosis [49]. Animal studies indicate that the inhibition of factor XII prevents thrombosis without exerting increased bleeding risk [50]. Activation of the contact system also leads to the production of inflammatory mediators, in particular bradykinin, a vasoactive substance with the potential to increase vascular leakage. The contact system may be activated in vivo by inorganic polyphosphates released from dense granules of activated platelets, or by neutrophil extracellular traps [51]. Interestingly, recent studies suggest that activated endothelial cells may also form a surface that is capable of activating the contact system [52].

Increased activity of the coagulation system in the course of inflammation results not only from direct activation of coagulation, but is also a consequence of the increase in plasma fibrinogen concentrations and increased expression of endothelial TF and P-selectin [31]. Moreover, inflammation leads to increased levels of C-reactive protein (CRP) and platelet activation with the exposure of procoagulant phospholipids [31,53]. Influence of CRP on coagulation is associated with facilitating interaction between monocytes and vascular endothelial cells [54], as well as with increased production of TF by monocytes [55] and thus activation of the extrinsic pathway [56]. Hypercoagulability observed in inflammation is also a result of inhibition of natural anticoagulants and decreased fibrinolytic activity. CRP increases the expression of plasminogen activator inhibitor-1 (PAI-1) by endothelial cells [57], while the production of prostacyclin is reduced [58]. This results in a reduction of the plasminogen activation and increased platelet aggregation.

Antithrombin inactivates thrombin as well as factors Xa, IXa, and VIIa linked with TF [31], resulting in the inhibition of fibrin formation. In the case of free antithrombin, this effect is weak; however, the thousand-fold increase of the inhibitory effect of antithrombin on thrombin and Xa occurs after complexing with heparin or glycosaminoglycans present on the surface of endothelial cells [47]. In the inflammatory state, accelerated degradation and inhibition of antithrombin activity occurs [31,59] as well as reduced expression and accelerated degradation of endothelial cells’ glycosaminoglycans [60].

Moreover, the anticoagulant pathway of protein C is inhibited in inflammation [31]. Endotoxin, IL-1β and TNF-α inhibit the expression of thrombomodulin and the endothelial cell protein C receptor, thus reducing the formation of activated protein C and inhibiting its anticoagulant activity [61,62].

## 3. Endothelial Cells at the Interface of Coagulation and Inflammation

Endothelial cells have several important functions above being a barrier between blood and tissues. They control vascular pressure and permeability, activation and adhesion of platelets and leukocytes, coagulation and fibrinolysis. Their precise characteristics differ between organs (with special characteristics of brain, lung and kidney vessels), and between venous and arterial vascular beds. Endothelium is a dynamic tissue, quickly responding to various stimuli [63].

Endothelial cells are important for both hemostasis and inflammation. Healthy endothelium under resting conditions exerts both anti-inflammatory and antithrombotic functions. Upon activation by inflammatory factors, such as IL-1β and TNF-α, endothelial cells increase expression of adhesion molecules, i.e., intercellular adhesion molecule-1 (ICAM-1), vascular cell adhesion molecule-1 (VCAM-1), and E- and P-selectins, leading to increased adhesion of leukocytes and platelets and transmigration of leukocytes through vascular wall. Inflammatory signals result in more procoagulant phenotype of endothelial cells, with TF and factor V expression, production of PAI-1, downregulation of thrombomodulin, and decrease of protein C synthesis [64]. Also, multiple stimuli, including thrombin, histamine, leukotrienes, superoxides, complement components (C5a, C5b-9), vascular endothelial growth factor (VEGF), vasopressin or epinephrine lead to degranulation of Weibel-Palade bodies and the release of large multimers of vWF as well as other proteins (including P-selectin, IL-8, eotaxin-3, angiopoietin-2, endothelin-1 and osteoprotegerin) [65].

Recently, the importance of angiogenic signaling pathways for vascular permeability became evident. Two main signaling systems are known, namely VEGFs/VEGF receptors and angiopoietins/Tie receptors. VEGF, also known as vascular permeability factor, is capable of strongly increasing vascular permeability [66]. In systemic inflammation, high concentrations of VEGF are observed in blood [67]. Angiopoietin-2 is stored in Weibel-Palade bodies and rapidly released upon stimulation; its binding to Tie-2 receptor leads to destabilization of the endothelium and increased vascular permeability [68].

## 4. Vascular Involvement in Acute Pancreatitis

In early pathogenesis of AP, microvascular abnormalities play an important role; in particular, SAP is associated with early impairment of pancreatic blood flow (reviewed in [23]). In mild AP, pancreatic capillary blood flow increases; however, SAP is associated with substantial early decrease in capillary blood flow, with complete capillary stasis observed in almost 40% of pancreatic capillaries [69].

Numerous clinical and experimental studies have shown that pancreatic ischemia plays an important role in the development of AP and in the progression of the disease to severe necrotizing pancreatitis [70,71,72]. As shown in a porcine model of severe AP, microcirculatory derangements are responsible for the decreased pancreatic tissue oxygenation and tissue damage, and the severity of microvascular disturbance is positively associated with mortality [73]. In AP evoked by pancreatic ischemia followed by reperfusion, disturbance of pancreatic blood flow is a primary cause of this disease. Numerous animal studies have indicated that pancreatic ischemia may be a causal factor in the pathogenesis of AP [71,72,74,75]. A vascular mechanism plays an essential role in the development of AP in some experimental settings [76]. Also, AP develops in clinical situations associated with ischemia of the pancreas, such as shock, cardiac surgery, or pancreatic transplantation [23,70]. In human necrotizing AP of various etiologies, microcirculatory derangements were confirmed by histopathological studies revealing microcirculatory intravascular thrombosis, intravascular stasis and endothelial desquamation, as well as parenchymal swelling of the pancreas thus reflecting the increased microvascular permeability preceding the development of pancreatic necrosis [77].

Moreover, improvement of pancreatic blood flow inhibits the development of AP and accelerates the recovery [78,79,80]. In recent experimental animal studies, where AP was induced by cerulein and ischemia/reperfusion, the course of acute pancreatitis was significantly milder after administration of digestive tract hormones such as obestatin and ghrelin [81,82,83]. Administration of these hormones improved pancreatic blood flow and, by their anti-inflammatory effect, led to acceleration of recovery from AP.

Microcirculatory changes in early SAP are not confined to the pancreas, but are also well documented in other organs, i.e., colon and ileum, liver, lungs, kidneys, heart and brain [84,85,86,87]. In the early phase of organ failure due to SAP (only 3 h post induction of AP by taurocholate in rats), edema, leukocyte adhesion to capillary walls and infiltration in tissues were observed in histopathological examination of liver, kidney, lung, intestine, and spleen. Areas of necrosis were detected in kidneys, intestine, spleen and lymph nodes. In pancreas, liver and kidneys, these changes were also accompanied by microvascular thrombosis [87]. Experiments using intravital microscopy confirmed increased capillary permeability and leukocyte rolling and sticking in distant organs in the early phase of SAP, leading to decreased blood flow velocity [84,86]. Recently, the disturbances and heterogeneity of capillary blood flow have been underscored as a cause of diminished blood oxygenation in lungs and oxygen supply to tissues, despite preserved total organ perfusion [88].

Several vasoactive substances have been associated with microcirculatory impairment in AP, including nitric oxide, bradykinin, endothelins, and platelet-activating factor (PAF) [89]. The role of nitric oxide in AP is still controversial and has been extensively reviewed elsewhere [90]. Other mediators were with some success targeted in experimental AP. In severe porcine AP and in various rat models of AP, pretreatment with the inhibitor of bradykinin B2 receptor (icatibant) showed beneficial effects [91,92,93,94]. Endothelin-1, a potent vasoconstrictor, was shown to be up-regulated in pancreatic endothelial cells by inflammatory stimuli, including cytokines, thrombin, and trypsin, which was associated with impaired splanchnic microcirculation in SAP [95]. Inhibition of endothelin receptor A attenuated reduced functional capillary density associated with experimental SAP in rats, and ameliorated platelet-endothelial and leukocyte-endothelial cell interactions, reducing numbers of stagnant platelets and leukocytes in pancreatic postcapillary venules [96]. PAF is a pleiotropic phospholipid mediator, with roles in hemostasis (platelet activation), endothelial cell activation (increase of capillary permeability), and inflammation (induction of cytokines, including IL-1β, TNF-α and IL-6) [97]. Several PAF receptor antagonists were beneficial in experimental AP as reviewed by Chen et al. [98]. Unfortunately, despite encouraging results of phase II human trials [99,100], in randomized phase III trial, PAF antagonist lexipafant did not prevent new organ failure or ameliorate systemic inflammatory response syndrome (SIRS) in patients with predicted SAP [101]. More recently, pretreatment with recombinant PAF acetylhydrolase was evaluated for the prevention of post-endoscopic retrograde cholangiopancreatography (post-ERCP) pancreatitis in randomized multicenter trial. No beneficial effects on the incidence or severity of AP was shown, despite high number of patients enrolled (600 patients) [102].

## 5. Laboratory Markers of Endothelial Activation and Injury in Acute Pancreatitis

Destabilization of the vascular endothelium, increased vascular permeability, disrupted vasomotor regulation, and activated coagulation lead to early complications of acute pancreatitis [103,104]. Fluid sequestration in patients with AP within the first 48 h from admission is significantly associated with SIRS criteria and the subsequent development of multiorgan failure [105].

VEGF is one of the most potent mediators capable of increasing vascular permeability. Several groups have studied the involvement of VEGF in AP. The results of both animal and human studies are, however, discrepant to some extent. Increased expression of VEGF was detected in the inflamed pancreas and associated with the increased vascular permeability observed in the early phase of AP [106,107]. The tyrosine kinase inhibitor of VEGF signaling attenuated almost completely the increased vascular permeability in the pancreas during experimental AP [107]. In rats with mild AP and severe necrotizing AP, serum VEGF concentrations were higher than in control animals [108]. However, infusion of VEGF in rats with SAP partially inhibited apoptosis in small intestine, kidney and liver, without affecting water content of the lung, the volume of ascitic fluid or hematocrit, suggesting a protective role of VEGF against endothelial injury in distant organs [108,109]. In the study of Ueda et al. [109], serum VEGF in the early phase of AP was higher among patients with moderately severe and severe AP, and was positively associated with kidney and liver failure, although not with mortality. Conversely, Mentula et al. [110] did not observe differences in VEGF concentrations between patients with AP who developed organ failure and those who did not.

A decoy VEGF receptor, soluble fms-like tyrosine kinase 1 (sFlt-1), has been strongly associated with the severity of sepsis [111]. We observed positive correlations between soluble sFlt-1 and the severity as well as complications of AP, such as acute kidney injury and activated coagulation [112].

Angiopoietin-2 has been proposed as a causative factor and a laboratory marker of endothelial cells’ destabilization and increased vascular permeability. In patients with AP, higher angiopoietin-2 predicted SAP, multiorgan failure, infectious complications and bowel ischemia as well as mortality [113,114]. Increased angiopoietin-2 was positively associated with the severity of AP, particularly kidney injury in the early phase of AP [104]. In recent years, angiopoietin-2 emerged as one of the most promising biomarkers for the early prediction of AP severity (Table 1).

Angiopoietin-2 is known to be stored in Weibel-Palade bodies of endothelial cells. The main protein of Weibel-Palade bodies, namely vWF, is also increased in plasma during SAP. In rats, SAP was associated with increased plasma vWF and soluble endothelial protein C receptor, as well as with increased endothelial cell apoptosis in the aorta as compared to the mild AP [115]. Increased plasma vWF was also reported in humans with SAP: its concentrations correlated positively with the severity of organ failure, APACHE III and sequential organ failure assessment (SOFA) scores, and significantly predicted acute lung injury [116] (Table 1). Several reports were published showing coincidence between AP and thrombotic thrombocytopenic purpura [117,118,119]. Morioka et al. [117] found highly increased concentrations of vWF (mean on admission 402%) coinciding with low activities of a disintegrin-like and metalloproteinase with thrombospondin type-1 motifs 13 (ADAMTS-13) among 13 SAP patients without disseminated intravascular coagulation (DIC). Mean ADAMTS-13 activity on admission was 37%, decreased to 32% on day 2 and subsequently increased. ADAMTS-13 activity negatively correlated with the APACHE II score as well as with markers of inflammation (IL-6, IL-8, fibrinogen, CRP, leukocytes) and pancreatic enzymes (serum amylase, elastase 1, trypsin and lipase). No ADAMTS-13 inhibitor was detected. Consequently, ultra large vWF multimers were detected in patients with the highest vWF/ADAMTS-13 ratios. 

Also, serum concentrations of osteoprotegerin, another protein stored and released from Weibel-Palade bodies upon endothelial activation, are significantly higher in patients with SAP as compared to mild AP [120].

Adhesion proteins, including E- and P-selectins, ICAM-1 and VCAM-1 have been studied as markers of endothelial activation or injury in AP. E-selectin is synthesized de novo by endothelial cells stimulated by IL-1, TNF-α, endotoxin and oxidative stress. Its soluble form occurs due to shedding from the surface of activated endothelial cells. P-selectin is stored in Weibel-Palade bodies, and thus may be rapidly released upon stimulation of endothelial cells, e.g., by thrombin or histamine. Additionally, upon stimulation by TNF-α and IL-1β, endothelial cells synthesize P-selectin. In experimental AP, prophylactic inhibition of P-selectin resulted in reduced platelet activation, platelet-endothelium and leukocyte-endothelium interactions and reduced pancreatic tissue inflammation and necrosis [127]. Both P- and E-selectins are overexpressed in lung tissue during experimental AP. Their up-regulation was associated with increased sequestration of neutrophils and pulmonary injury observed in histological examination [128]. ICAM-1 is constitutively expressed by endothelial cells, but the expression highly increases upon inflammatory stimulation. VCAM-1 expression is specific to endothelium; together with ICAM-1, it is involved in leukocyte adhesion and rolling. Increased ICAM-1 expression was reported in lungs of rats with SAP [129]. Blocking ICAM-1 with an antibody resulted in reduced neutrophil sequestration, decreased microvascular permeability and improved lung histology [129]. Frossard et al. [130] also found increased ICAM-1 in serum, pancreas and lungs of mice with AP induced with cerulein or with choline-deficient, ethionine-rich diet. Both pancreatitis and lung injury were diminished but not completely prevented in mice with ICAM-1 deficiency. In porcine model of SAP, increased expression of adhesion proteins was shown: platelet-endothelial cell adhesion molecule-1 in liver, kidney and pancreas, VCAM-1 in kidney, and P-selectin in liver [131].

Increased soluble E-selectin has been proposed as a marker of severe AP in several studies (Table 1). Wereszczynska-Siemiatkowska et al. [122] reported increased soluble E-selectin during first 10 days from admission among patients with SAP, as compared to those with mild AP and to patients with non-AP acute abdominal pain (mainly acute biliary tract diseases). At admission of AP patients, strong correlations were observed between soluble E-selectin and IL-6 concentrations, polymorphonuclear elastase activity, as well as oxidative stress markers (serum malondialdehyde and 4-hydroxyalkenals) [122]. Of note, patients with severe AP had also increased IL-10 serum concentrations, especially during the first two days from admission, and positive correlation was found between E-selectin and IL-10 [122]. In another study, soluble E-selectin, ICAM-1, TF and vWF were shown to be significantly higher in SAP associated with acute respiratory distress syndrome; all studied endothelial markers correlated positively with APACHE III and SOFA scores, and negatively with oxygenation index (PaO_2_/FiO_2_) during the first 10 days of hospital stay due to AP [116]. Also, Powell et al. [132] and Ida et al. [126] reported higher soluble E-selectin in patients with SAP, especially in those who subsequently died, as compared to mild AP. Moreover, during 3 days from admission, E-selectin concentrations were increasing in severe disease in contrast to mild AP [132]. Nakae et al. [133] observed positive correlation between soluble E-selectin and TNF-α in the early phase of human AP; both mediators were positively associated with AP severity. Hynninen et al. [134] reported similarly increased soluble E-selectin in patients with SAP and with severe sepsis, positively correlated with SOFA scores. However, there are also contradictory reports. It was suggested that the peak soluble E-selectin concentrations are observed late (after 72 h from the onset of AP symptoms) and can therefore not be used as an early marker of AP severity [135,136]. During first 6 h from the onset of pain, Pezzilli et al. [123] did not observe higher soluble E-selectin in AP patients compared to healthy controls; however, it was higher in SAP than in mild AP. Ida et al. [126] did not show significant difference in soluble E-selectin on admission and on subsequent days in those who died from AP compared to survivors. Nonetheless, both mild and severe AP were associated with concentrations above the reference limit.

Reports regarding soluble P-selectin in patients with AP are scarce. In a small study of Powell et al. [132], serum soluble P-selectin concentrations during 3 days from admission did not differ between mild and severe AP, but were significantly higher in non-survivors than survivors. To the contrary, Pezzilli et al. [123] reported lower soluble P-selectin in SAP patients’ sera as compared to those with mild AP and healthy controls.

Soluble ICAM-1 has been associated with the severity of human AP (Table 1). Pezzilli et al. [123] observed higher concentrations of soluble ICAM-1 in patients with SAP versus mild AP. This result is, however, depreciated by the fact that the levels in patients with AP were not significantly different compared to healthy volunteers. Siemiatkowski et al. [116] have shown that soluble ICAM-1 may serve as a marker of AP-associated lung injury. In this study, strong positive correlations were observed between plasma-soluble ICAM-1 and severity of organ dysfunction (APACHE III and SOFA scores) in SAP patients. Nakae et al. [133] observed higher soluble ICAM-1 in patients who died from AP than in survivors.

Studies of soluble VCAM-1 in patients with AP are inconclusive. Serum VCAM-1 in AP patients on admission was lower than in controls and did not correlate with AP severity in one study [123], while it was higher among non-survivors of SAP and positively correlated with ICAM-1 and TNF-α in another study [133].

Elevated plasma concentrations of thrombomodulin in inflammation are caused by shedding of membrane-bound thrombomodulin from endothelial cells by neutrophil elastase. In a small study, Ida et al. [126] have shown that increased plasma concentrations of soluble thrombomodulin was positively associated with the severity of AP and was higher in patients who died. Mantke et al. [124] studied soluble thrombomodulin during the first 28 days after the onset of symptoms of AP: starting from day 3, non-survivors had significantly higher concentrations than survivors. The clinical studies consistently reported positive association between plasma thrombomodulin in the early phase of AP and more severe disease [124,125,126] (Table 1). Plasma tissue factor pathway inhibitor (TFPI) in human AP was shown to be higher in patients with SAP as compared to mild AP, and was positively correlated with inflammatory mediators, thrombomodulin and PAI-1, consistent with the assumption that plasma TFPI levels reflect endothelial injury rather than anticoagulation [137].

Recently, levels of endothelial-specific microRNAs (miR-551-5p and miR-126a-5p) were associated with the severity of human AP [138].

## 6. Disturbances of Hemostasis in Relation to Inflammation in Acute Pancreatitis

In experimental and human AP, abnormalities were reported regarding all aspects of hemostasis [26]. Decreased numbers of platelets and increased platelet activation were observed in the early phase of AP [139,140,141,142,143]. Plasma TF concentrations were increased [116,144,145]. The levels of prothrombin, fibrinogen and factor X gradually decreased [146], and prolonged clotting times (prothrombin time, activated partial thromboplastin time and thrombin time) were observed [139,146,147]. Decreased concentrations of natural anticoagulants, especially protein C and antithrombin, were consistently reported [139,148,149,150]. Activity of tissue plasminogen activator (tPA) and PAI-1 was increased [151,152]. A complex of α2-plasmin inhibitor with plasmin was increased in patients with the most severe AP [142]. These changes are consistent with the activation of the coagulation system, following local and systemic inflammation, leading to consumptive coagulopathy. The degree of coagulation abnormalities in AP depends on the severity of inflammation [146,147]. In mild pancreatitis, thrombosis may be limited to pancreatic microcirculation. In severe systemic inflammation, DIC may occur [139,153]. Activation of fibrinolysis secondary to activated coagulation results in increased concentrations of fibrin/fibrinogen degradation products, including d-dimer, that are significantly correlated with inflammatory markers and AP severity [142,143,147,150,154].

The classic Virchow’s triad of factors predisposing to thrombosis, i.e., procoagulant changes in the blood components, procoagulant properties of the vessel wall and decreased blood flow velocity, can be observed in SAP. Consequently, various clinically relevant thrombotic complications are observed in human AP, ranging from localized thrombosis [155,156,157,158] and pulmonary thromboembolism [159] to DIC [153,160]. Both thrombotic and hemorrhagic complications were associated with deaths due to AP [27].

Even in the absence of clinically significant thrombotic complications, laboratory tests reveal the activation of coagulation and fibrinolysis, related to the severity of AP. Maeda et al. [142] reported significant correlations between laboratory parameters of DIC and the severity of AP measured in the five-stage Japanese scoring system: more severe AP was associated with lower platelet counts and antithrombin concentrations as well as higher levels of d-dimer, fibrin/fibrinogen degradation product E, and thrombin-antithrombin complexes. All the parameters were also significantly associated with mortality (Table 2); and the diagnosis of DIC was much more prevalent among patients who died (79% versus 10% of patients) [142]. Also, longer PT, APTT, higher fibrinogen and d-dimer, lower protein C and antithrombin, lower plasminogen, and higher PAI-1 on admission and 24 h thereafter were associated with organ failure (pulmonary, kidney or cardiovascular) in the course of AP in humans [161] (Table 2). Of note, high d-dimer concentrations in SAP were observed already at admission (1–2 days from the onset of symptoms), but persisted during the subsequent week and even during remission of AP [143,147,150]. Also, patients with moderately severe AP were characterized by higher d-dimer and lower protein C levels as compared with mild AP [162]. Plasma TF concentrations were significantly increased in SAP during the first 10 days from admission, and significantly correlated with APACHE III and SOFA scores as well as with PaO_2_/FiO_2_ [116]. The admission TF concentrations significantly predicted AP-associated lung injury [116] (Table 2). In patients who eventually died from AP as compared to patients who survived, natural anticoagulants (antithrombin and protein C, but not protein S) were lower, and increased levels of PAI-1 and d-dimer were observed during the preceding period [150,161,163]. In patients with SAP as defined by the original 1992 Atlanta classification (organ failure and/or local complications), the development of organ failure was associated with decreased protein C concentrations as well as decreased activated protein C [148]. Protein C and activated protein C levels correlated with the numbers of activated monocytes [148].

The results of the studies that reported diagnostic accuracy of laboratory markers of hemostasis are summarized in Table 2. Most reliable diagnostic accuracy was consistently reported for d-dimer measured at admission for the prediction of (multi)organ failure [161,164] and antithrombin for the prediction of death [142] (Table 2).

On the other hand, activated platelets and coagulation have been shown to drive inflammation in AP. Platelets enhance leukocyte rolling, sticking and transmigration in pancreatic venules in the early phase of AP [96,166]. Platelet P-selectin appears crucial for leukocyte recruitment and rolling in inflamed venules of the pancreas [167,168]. PAR-2 signaling was implicated in AP pathogenesis; its inhibition protected mice against experimental biliary pancreatitis and AP-associated lung injury [169,170].

These observations have led to the assumption that anticoagulant or antithrombotic treatment in AP may result not only in reduction of (micro) thrombosis and improved microcirculation but also in reduced local and systemic inflammation.

## 7. Therapeutic Effects of Anticoagulants in Acute Pancreatitis

### 7.1. Heparin

Heparin as a cofactor of antithrombin inhibits thrombin activity as well as factor Xa activity and thus thrombin formation. Heparin attenuates not only procoagulant but also proinflammatory effects of thrombin. Moreover, heparin in a complex with antithrombin or heparin cofactor II is able to reduce activity of trypsin and chymotrypsin as well as the conversion of trypsinogen into trypsin [171,172]. Various experimental studies have shown the anti-inflammatory effect of heparin, administered both in a protective and therapeutic manner in AP [171,173,174,175,176,177,178]. Earlier studies have been summarized by Hackert et al. in 2004 [179].

In humans, several trials explored the utility of unfractioned or low molecular weight heparin (LMWH) to prevent post-ERCP pancreatitis in high-risk patients [180,181,182,183]. Neither of the studies demonstrated reduced AP or SAP ratios in the treatment groups. A meta-analysis [177] performed in 2011 that included the four trials cited above (1438 patients in total) did not show significant benefit of prophylactic heparin in prevention of post-ERCP pancreatitis (relative risk 0.67, 95% confidence interval 0.44–1.03, *p* = 0.07) or post-ERCP SAP (relative risk 0.62, 95% confidence interval 0.15–2.60, *p* = 0.51). There were no differences between unfractioned and low molecular weight heparin. Of note, no increased bleeding risk was shown as well (relative risk for ERCP-related hemorrhage 0.84, 95% confidence interval 0.34–2.03, *p* = 0.69).

Heparin has been used in the treatment of SAP caused by severe hypertriglyceridemia. Such treatment is justified by the ability of heparin to stimulate lipoprotein lipase activity [184]. Several case reports or case series suggested effectiveness of heparin, usually administered in conjunction with insulin, in lowering triglyceride concentrations in such patients [184,185,186,187]. The results of a clinical trial evaluating the effects of LMWH and intensive insulin therapy in SAP were published in 2014 [188]. The trial included 134 adult patients with SAP treated in single-center (General Hospital of PLA, Beijing, China), randomly assigned to four groups: control group treated conventionally, intensive insulin therapy group, LMWH group (5000 U every 12 h) and combined treatment group (insulin plus LMWH), in addition to conventional therapy. Authors reported reduced lengths of stay, incidence of multiorgan failure, need for surgery and mortality in treatment groups, with best results of combined treatment. Four patients (12%) died in the control group (conventional treatment), as compared to one death (3%) in the intensive insulin therapy group, one death (3%) in the LMWH group and no deaths in the group administered combined therapy.

The use of LMWH for treatment of SAP was also evaluated in a multicenter randomized trial that recruited 265 patients from four hospitals from China [189,190]. LMWH was administered in dose 100 µg/kg/day starting at admission, until day 7 of the hospital stay. Balthazar computed that the tomography scores at the end of the first and second week of the hospital stay were better in the treatment group than in the control group (conservative treatment), as well as APACHE II score for week 2. The incidence of acute respiratory distress syndrome, pancreatic encephalopathy, multiorgan failure, and mortality (10.4% versus 30.6%) was lower in the treatment group.

### 7.2. Activated Protein C

In experimental SAP, treatment with activated protein C (APC) resulted in decreased inflammation (decreased expression of pancreatic TNF-α and IL-1β proteins, decreased serum TNF-α, IL-8 and IL-6), increase in pancreatic expression of endothelial protein C receptor and thrombomodulin, and reduced severity of pancreatic morphological changes, including necrosis [149,191,192]. Bacterial translocation to mesenteric lymph nodes and to pancreas was reduced in APC-treated rats with SAP [192]. However, a contradictory report was also published, illustrating that administration of APC did not result in improved histopathologic scores of the pancreas and, in fact, was associated with significantly higher serum IL-6 [193]. In the study of Alsfasser et al. [194], despite no difference in the histopathologic scores of the pancreas, rats with SAP that were treated with APC presented reduced pancreatic and pulmonary inflammation (reduced myeloperoxidase activity) and improved survival.

A small clinical trial was undertaken to evaluate safety and efficacy of the treatment with APC in AP (APCAP study) [195]. A prospective double-blind randomized study included 32 patients with SAP and no sepsis from a single center (Helsinki University Central Hospital, Helsinki, Finland). Patients were admitted within 96 h from the onset of pain. APC was administered intravenously for 96 h in a fixed dose of 24 μg/kg/h; physiologic saline was used as placebo. All patients received the treatment according to the initial randomization. No significant difference was observed between treatment and placebo groups regarding the primary efficacy endpoint, i.e., the change in SOFA score between the start of the study drug (day 0) and day 5. In fact, a small non-significant difference in advantage of placebo was found. Three deaths due to multiple organ failure occurred in the treatment group and none in the placebo group (autopsy excluded bleeding as a cause of the deaths). The only significant difference was the increase in total and conjugated bilirubin in the treatment group. No serious bleeding occurred in treated patients; four patients had minor bleeding (from mouth, nose, or urinary tract) versus two patients in the placebo group. Recent analysis revealed that APC did not alleviate coagulative disorders in patients included in the APCAP study; rather, the treatment with APC was associated with retarded recovery from coagulopathy [196].

### 7.3. Soluble Thrombomodulin

Eguchi et al. [197] performed a retrospective analysis of 54 adult patients with SAP diagnosed according to Japanese severity scoring system, treated in a single center (Osaka Saiseikai Nakatsu Hospital, Osaka, Japan), of whom 24 developed DIC and were therefore treated with recombinant human soluble thrombomodulin (rTM). The study included patients in whom treatment started within the first 48 h from the onset of pain. Patients who were subsequently treated with rTM had on average more severe disease, with higher APACHE II and SOFA scores on admission, i.e., before treatment. Acute necrotizing collections within the pancreas were equally prevalent in both treated and untreated groups at admission. rTM was administered in dose 380 or 130 U/ kg/day for patients on hemodialysis. The treatment was introduced in those who were diagnosed with DIC and continued until remission of DIC. Other aspects of AP treatment did not differ between the groups. The length of hospital stay, need for intensive care, length of intensive care unit stay, incidence of persistent organ failure, or mortality did not differ between the groups. Walled-off necrosis at 4 weeks from admission or later was significantly less prevalent in the treated group (29% versus 57% of patients). No serious adverse events (e.g., bleeding) were recorded in the treatment group.

### 7.4. Other Anticoagulants

In a recent experimental study, pretreatment with low doses of acenocoumarol, a vitamin K antagonist, attenuated ischemia/reperfusion-induced AP and was associated with reduced leukocyte inflammatory infiltration of the pancreas as well as diminished pancreatitis-induced increase in serum IL-1β concentrations [198]. The results were confirmed in another model of experimental AP, i.e., cerulein-induced AP in rats [199]. Since acenocoumarol is a commonly used antithrombotic drug with well-known safety profile, its usefulness in prevention of AP among patients with pancreatic circulation disorders would be worth studying.

Pretreatment with antithrombin was shown to ameliorate cerulein-induced AP in rats [200]. Edema, inflammation and necrosis of the pancreas were partially reduced and serum concentrations of IL-6, TNF-α, and high-mobility box group 1 protein were diminished in mice pretreated with antithrombin. Similar results were obtained with pretreatment with low molecular weight heparinoid, danaparoid sodium, that was also shown to inhibit cerulein-induced NFκB activation [201].

Andersson et al. [202] evaluated the effects of pretreatment with active side-inactivated factor VIIa in rats. AP was then induced by infusion of sodium taurodeoxycholate into common bile duct. Myeloperoxidase activity was significantly reduced in ileum and lungs of pretreated animals, and serum concentrations of inflammatory markers were lowered.

## 8. Conclusions

The interplay between inflammation, coagulation and endothelial activation is involved in the earliest local events in acute pancreatitis (AP), and is associated with the early phase of systemic disease in severe AP (SAP), although many aspects remain unknown. From the current evidence on this subject, there are several practical conclusions: (1) Systemic inflammation as seen in SAP is not rarely associated with thrombotic disorders, and the activation of coagulation may further aggravate inflammation. Laboratory tests in SAP often reveal abnormalities of coagulation, while clinically relevant disorders of coagulation in AP are associated with significantly worse prognosis; (2) Markers of coagulation/fibrinolysis measured early in the course of AP, in particular d-dimer, are significantly associated with AP severity, and may therefore be used to assist treatment decisions and prognosis; (3) Markers of endothelial dysfunction, in particular angiopoietin-2, may prove even more useful; however, we need robust, routine and standardized laboratory tests, currently available only for sFlt-1; (4) There are severe difficulties in translation between animal experiments using anticoagulant or antithrombotic medications and their use in humans. There may be several reasons. Patients with AP form a much more heterogeneous group than the experimental animals, both regarding the causes of AP and the time from the disease onset to the start of treatment. In substantial proportion of patients, systemic inflammation and organ failure are present already at admission. Also, there are difficulties in recruiting enough patients for clinical trials; (5) Nevertheless, some reports from clinical trials with low molecular weight heparin treatment are promising. However, the results have to be corroborated by other groups before this treatment can be recommended; (6) Although the use of anticoagulants was rarely associated with significant benefits in clinical trials, there were also no significant bleeding complications. This fact encourages the use of such drugs in the treatment of thrombotic complications of AP.

Further clarification of the relationships between inflammation, pancreatic blood flow, coagulation system, endothelial involvement and the development and course of AP is needed. Several anticoagulant or antithrombotic drugs have ameliorated AP severity in experimental designs, and their therapeutic potential is still worth being tested in patients.

## Figures and Tables

**Table 1:** Diagnostic accuracy of laboratory markers of endothelial activation or injury measured in serum or plasma for the prediction of severity, complications and mortality of acute pancreatitis (AP).

Laboratory Test	Studied Group	Time of Blood Collection	Outcome Variable (Number of Cases)	Values Associated with Outcome Variable	Se., % ^1^	Sp., % ^2^	AUC ^3^	Ref. ^4^
Angiopoietin-2	28 patients with AP from University of Pittsburgh Medical Center	Within 3 days from the onset of pain ^5^	Severe AP (persistent organ failure >48 h or death) (6 patients form Pittsburg and 14 from Greifswald)	>1.91 ng/mL	83	91	0.940	[113]
	123 patients with AP from Greifswald University			>2.94 ng/mL	93	63	0.790	
	25 patients with AP	At 12 h from admission (admission within 72 h from the onset of pain)	Severe AP according to 1992 Atlanta classification (7 patients)	>10 ng/mL	100	88	0.970	[121]
	115 patients with AP (subsample from PROPATRIA trial cohort)	Within 5 days from admission (median 3 days from the onset of pain)	Severe AP (organ failure or pancreatic necrosis) (37 patients)	>4.56 ng/mL	81.1	73.2	0.851	[114]
			Multiorgan failure (18 patients)	>5.01 ng/mL	72.2	73.2	0.784	
			Infectious complications of AP (39 patients)	>4.51 ng/mL	79.5	76.3	0.816	
Soluble fms-like tyrosine kinase 1	66 consecutive adult patients with AP	At 24 h from the onset of pain	Severe and moderately severe AP according to 2012 Atlanta classification (20 patients)	>139 pg/mL	94%	63%	0.808	[112]
		At 48 h from the onset of pain		>120 pg/mL	78	77	0.791	
Soluble E-selectin	56 patients with AP	At admission (≤48 h from the onset of pain)	Severe AP according to Ranson’s score and Balthazar CT grading (28 patients)	increased	NR ^6^	NR ^6^	0.802	[122]
	15 consecutive patients with AP	At admission (≤6 h from the onset of pain) and on two subsequent days (pooled results)	Severe AP according to 1992 Atlanta classification (5 patients)	>3.92 ng/mL	60	90	0.780	[123]
	69 adult patients with severe AP	At admission (≤48 h from the onset of pain)	Acute respiratory distress syndrome in the course of severe AP (39 patients)	>165.6 ng/mL	48.3	86.7	0.704	[116]
Soluble ICAM-1	15 consecutive patients with AP	At admission (≤6 h from the onset of pain) and on two subsequent days (pooled results)	Severe AP according to 1992 Atlanta classification (5 patients)	>80.4 ng/mL	73.3	70	0.684	[123]
	69 adult patients with severe AP	At admission (≤48 h from the onset of pain)	Acute respiratory distress syndrome in the course of severe AP (39 patients)	>711.2 ng/mL	61.5	93.3	0.787	[116]
Soluble thrombomodulin	73 patients with AP	On day 3 from the onset of pain	Death (12 patients)	<75 ng/mL	100	77	NR ^6^	[124]
	104 patients with AP	At 48 h from the onset of pain	Pancreatic necrosis (32 patients)	>71.5 µg/L	75	99	0.949	[125]
	27 patients with AP	At admission	Death (5 patients)	>32 TU/mL	80	91	0.876	[126]
von Willebrand factor (antigen)	69 adult patients with severe AP	At admission (≤48 h from the onset of pain)	Acute respiratory distress syndrome in the course of severe AP (39 patients)	>169.2%	43.2	93.3	0.686	[116]

^1^ diagnostic sensitivity; ^2^ diagnostic specificity; ^3^ area under receiver operating characteristic curve; ^4^ reference number; ^5^ the onset of pain due to AP is considered the onset of the disease; ^6^ not reported.

**Table 2:** Diagnostic accuracy of laboratory markers of hemostasis measured in whole blood (platelet count) or plasma (other markers) for the prediction of severity, complications and mortality of acute pancreatitis (AP).

Laboratory Test	Studied Group	Time of Blood Collection	Outcome Variable (Number of Cases)	Values Associated with Outcome Variable	Se., % ^1^	Sp., % ^2^	AUC ^3^	Ref. ^4^
Platelet count	139 consecutive patients with AP	At admission	Death (14 patients)	<92 × 10^3^/µL	75	71	0.850	[142]
d-dimer	139 consecutive patients with AP	At admission	Death (14 patients)	>6.1 µg/mL	85	67	0.783	[142]
	91 consecutive patients with AP	At admission	Organ failure: pulmonary or kidney failure, or shock (24 patients)	>0.414 µg/mL	90	89	0.908	[161]
		24 h from admission		>0.551 µg/mL	90	81	0.916	
	38 consecutive patients with AP	At admission	Organ failure (23 patients)	>0.4 µg/mL	81.7	54.2	0.683	[150]
			Death (14 patients)	>0.4 µg/mL	90.9	58.3	0.708	
	45 consecutive adult patients with severe AP	Day 0–2 from admission (mean value)	Multiorgan dysfunction syndrome (16 patients)	>0.812 µg/mL	81	90	0.899	[154]
			Pancreatic infection (14 patients)	>0.762 µg/mL	100	87	0.968	
		Day 0–2 from admission (maximum value)	Multiorgan dysfunction syndrome (16 patients)	>0.975 µg/mL	81	79	0.885	
			Pancreatic infection (14 patients)	>0.975 µg/mL	93	81	0.935	
	36 pediatric patients with AP (aged 1–17 years)	At admission	Multiorgan failure (4 patients)	>1.189 µg/mL	100	87.5	0.914	[164]
	173 adult patients with AP	At admission (≤96 h from the onset of pain ^5^)	Critical AP (persistent organ failure plus infected necrosis) (47 patients)	>0.67 µg/mL	83	68	0.810	[165]
	106 patients with mild to moderately severe AP	Within 24 h from admission (≤48 h from the onset of pain)	Moderately severe AP according to 2012 Atlanta classification	>0.91 µg/mL	62.2	84.1	0.747	[162]
Fibrin/fibrinogen degradation product-E	139 consecutive patients with AP	At admission	Death (14 patients)	>894 ng/mL	93	73	0.873	[142]
Antithrombin	139 consecutive patients with AP	At admission	Death (14 patients)	<69%	81	86	0.926	[142]
	91 consecutive patients with AP	24 h from admission	Organ failure: pulmonary or kidney failure, or shock (24 patients)	<75.5%	62	89	0.770	[161]
	38 consecutive patients with AP	At admission	Organ failure (23 patients)	≤71%	66.7	78.6	0.748	[150]
			Death (14 patients)	≤71%	70.8	81.8	0.830	
Protein C	38 consecutive patients with AP	At admission	Organ failure (23 patients)	≤60%	62.5	64.3	0.683	[150]
			Death (14 patients)	≤60%	70.8	63.6	0.691	
Thrombin-antithrombin complex	139 consecutive patients with AP	At admission	Death (14 patients)	>11 ng/mL	79	72	0.768	[142]
Tissue factor	19 patients with alcoholic SAP	At admission (≤48 h from the onset of pain)	Pancreatic necrosis (11 patients)	>350 pg/mL	60	100	0.773	[144]
	48 consecutive patients with AP	At inclusion into study (median duration of pain 34 and 25 h in mild and severe AP)	Severe AP according to 1992 Atlanta classification (21 patients)	>32 pg/mL	86	48	0.679	[145]
				>46 pg/mL	62	74		
	69 adult patients with severe AP	At admission (≤48 h from the onset of pain)	Acute respiratory distress syndrome in the course of severe AP (39 patients)	>168.4 pg/mL	61.1	90.0	0.757	[116]

^1^ diagnostic sensitivity; ^2^ diagnostic specificity; ^3^ area under receiver operating characteristic curve; ^4^ reference number; ^5^ the onset of pain due to AP is considered the onset of the disease.

## Abbreviations

AP	acute pancreatitis
NFκB	nuclear factor κB
SAP	severe acute pancreatitis
TF	tissue factor
PAR	protease-activated receptor
sCD40L	soluble CD40 ligand
TNF	tumor necrosis factor
vWF	von Willebrand factor
IL	interleukin
CRP	C-reactive protein
PAI-1	plasminogen activator inhibitor-1
ICAM-1	intercellular adhesion molecule-1
VCAM-1	vascular cell adhesion molecule-1
VEGF	vascular endothelial growth factor
PAF	platelet activating factor
ERCP	endoscopic retrograde cholangiopancreatography
SIRS	systemic inflammatory response syndrome
sFlt-1	soluble fms-like tyrosine kinase 1
APACHE	acute physiology and chronic health evaluation
SOFA	sequential organ failure assessment
ADAMTS-13	disintegrin-like and metalloproteinase with thrombospondin type-1 motifs 13
DIC	disseminated intravascular coagulation
CT	computed tomography
TFPI	tissue factor pathway inhibitor
tPA	tissue plasminogen activator
LMWH	low molecular weight heparin
APC	activated protein C
rTM	recombinant human soluble thrombomodulin
